# A unique case of suicide by crossbow with indirect triggering and cranial injury: forensic issues and literature review

**DOI:** 10.1007/s12024-024-00833-4

**Published:** 2024-05-28

**Authors:** Tambuzzi Stefano, Gentile Guendalina, Primavera Riccardo, Zoja Riccardo

**Affiliations:** Laboratorio di Istopatologia Forense e Microbiologia Medico Legale - Sezione di Medicina Legale e delle Assicurazioni - Dipartimento di Scienze Biomediche per la Salute - Università degli Studi di Milano, Via Luigi Mangiagalli, Milano, 37-20133 Italy

**Keywords:** Forensic pathology, Suicide, Crossbow, Autopsy, Intracranial injury, Bone injury

## Abstract

Currently, crossbows are involved in some deaths, including suicides. These are rare events for which an accurate study of the body discovery site and reconstruction of the triggering mechanism of the crossbow represent crucial medicolegal elements. In this report, a unique case of suicide by crossbow is presented, in which the male victim constructed an elaborate scenario. He arranged two tripod stands to hold the crossbow at the height of his head, and a third to support a hollow tube positioned in front of the muzzle of the crossbow to direct the bolt. After positioning the center of the forehead in front of the hollow tube, the trigger was activated from a distance using a hooked metal rod. The methods used prompted a literature review on suicide by crossbow, which revealed only 14 reports from 1993 to 2023. The head and chest were the main target areas, and in almost all cases, the victims directly pulled the trigger with their finger. Only one case of indirect triggering was found, with the chest as the target. Therefore, in this scenario, the case presented stands as a unique report, due to the elaborate system devised to carry out the suicide and accurately strike the predetermined target.

## Introduction

The crossbow, an ancient weapon [1] originating in China between the 8th and 5th centuries BC, played a crucial role as a defensive and offensive weapon in medieval military history [2], due to its accuracy and power. Over the years, it has been replaced by firearms [3], but it is still used today in competitive sports, such as hunting and target shooting [4]. It has a firm grip, long range, and is easy to operate without the requirement for specific training or strength, although its weight affects manoeuvrability and accuracy of shooting. Structurally, it consists of two rigid limbs attached to either end of the stock, a riser connecting the stock centrally to the limbs. A bowstring is attached to the tip of each limb, which is held under tension when drawn by the triggering mechanism, allowing a bolt to be launched when the trigger is pulled. The bolts, which are usually cylindrical in shape, are metal, carbon, wood, or fiberglass and have two to five vanes at the rear to impart the necessary rotation to provide stability during free flight [5]. In Italy, the crossbow is sports equipment and can be freely purchased by anyone over 18 years of age without the need for an ownership permit or notification to the authorities. However, the crossbow is a highly injurious and potentially lethal tool, particularly when used at close range, as it can launch a bolt at approximately 60 m/s with a high penetrating force capable of piercing even the most resistant structures such as bones [6]. Therefore, although it is an unusual weapon, it is known to be involved in deaths in modern times, raising significant interpretative challenges in distinguishing between accidents, homicide, and suicide. The study of the body discovery scene and reconstruction of the triggering mechanism should be the main pillars of this type of assessment. In this report, we present a unique case of a suicide by crossbow, characterized by an extremely unusual triggering method. We present the findings and forensic considerations and present a literature review on suicide by crossbow, with particular attention to the triggering mechanism.

## The case

### On-site judicial inspection

**Fig. 1:**
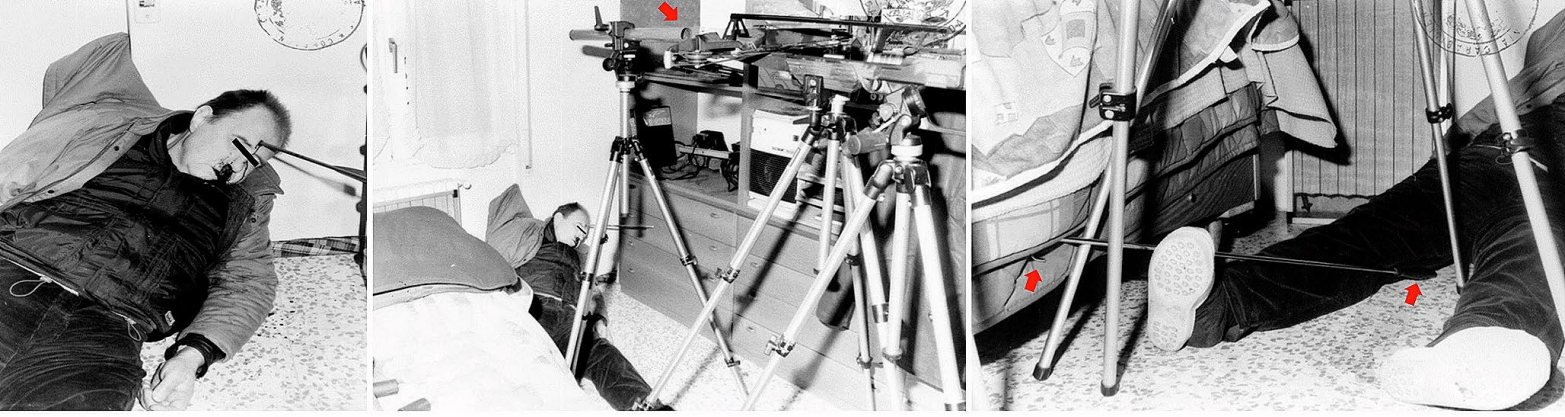
Pictures of the judicial inspection. On the left, a macroscopic view of the victim’s body. In the centre, the double tripod with the hollow metal tube (arrow) placed in front of the crossbow’s muzzle to guide the bolt with maximum precision to the target. On the right, the hooked metal rod (arrows) found near the body

A 56-year-old male was discovered dead in his locked house, semi-reclined on the floor, with a crossbow bolt lodged in his head. He lived alone, and at the time of death, no one else was known to be in the house, and there were no signs of forced entry. During the forensic examination, in front of the body, at approximately 60 cm, a Barnett® precision crossbow was mounted on two tripod stands, one in front of the other. In front of the crossbow muzzle, a cylindrical hollow metal tube was inclined slightly downward, supported by an additional tripod stand. The victim was lying semi-reclined on the floor, with his shoulders and head resting against the wall, and his legs stretched out under the tripods. A metal rod with a hook-shaped end was observed close to the back of the right foot. This end was in contact with the neighboring bedsheet, whereas the other end, with a black rubber grip, rested on the floor and was in contact with the right thigh. The rod was 55 cm in length and appeared clean and intact. At the conclusion of the on-site investigation, all equipment discovered at the scene was seized, and fingerprint analysis was performed on the hooked metal rod (Fig. 1).

### Gross examination

**Fig. 2:**
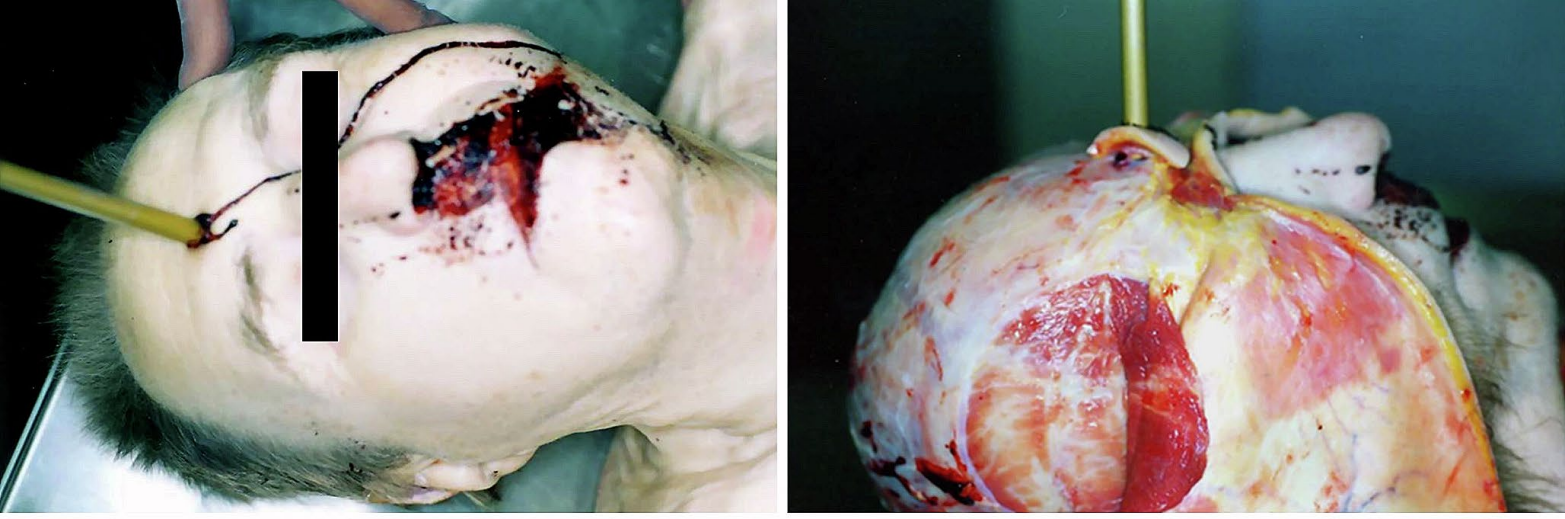
Macroscopic views during the autopsy, showing the penetrating injury in the centre of the forehead with the crossbow bolt still in place

**Fig. 3:**
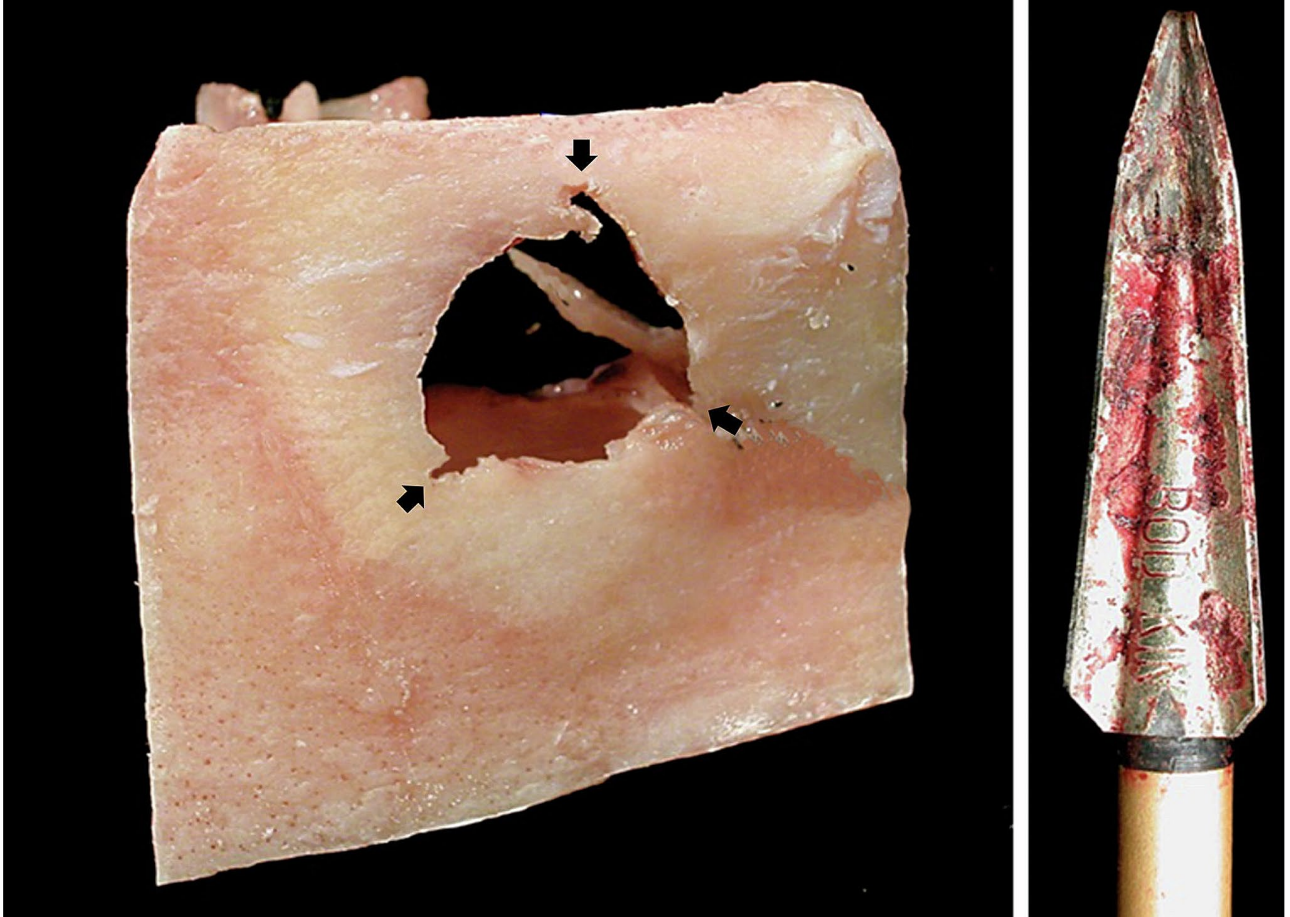
On the left, macroscopic view of the portion of frontal bone hit by the crossbow bolt, showing the three notches corresponding to the bolthead barbs (arrows), and on the right, a detail of the triple-sectioned metal tip of the bolt after extraction

A judicial autopsy was performed two days after discovery. The clothes did not exhibit any specific features. On external examination, the only observed traumatic finding was the presence of a bolt lodged in the center of the forehead, 2 cm above the glabella. A small entry wound with sharp inward margins infiltrated with blood was observed. A trail of coagulated blood was observed at the entry wound, and dried blood was observed in the nasal and perioral regions (Fig. 2). No exit wounds were observed. As the bolt was firmly embedded in the head, the skull was opened with the bolt in situ. After skull removal, the bolt was gently extracted, measuring 40.5 cm in length. It had a metal triple-sectioned barbed tip with a diameter of 1.5 cm, identified as a Bodkin®. The bolt penetrated the skull by approximately 20 cm and stopped at the right occipital bone without injuring it. A roughly circular-shaped fracture with jagged margins and multiple bone fragments was observed in the frontal bone. Upon closer examination, three notches were observed at different points on the fracture margin, corresponding to the barbs of the bolthead. A roughly triangular shape was formed by mentally connecting the three points, which was consistent with the bolthead section. Consequently, multifragmentation was observed with the loss of bone substance close to the impact point. Radial fracture lines were not observed around the site of bone injury (Fig. 3). Internally, a slightly oblique destructive anteroposterior penetrating pathway was observed, with perforation and damage to the meninges, right frontal lobe, olfactory and oculomotor nerves, anterior clinoid process, right cerebellar lobe, and brainstem. Multiple small bone fragments were observed along the intraparenchymal path.

At the conclusion of the autopsy, the cause of death was identified as cranioencephalic injuries caused by a penetrating head wound from a crossbow bolt.

The results of the fingerprint analysis were obtained, which only revealed prints attributable to the victim, particularly those of the right-hand on the grip of the hooked rod.

As a final assessment, it was noted that the combined length of the victim’s right upper limb and the hooked metal rod was sufficient to reach the trigger if he had been standing in front of the hollow tube facing the crossbow.

### Reconstruction of events

By integrating the autopsy findings with the results obtained from the onsite investigation, it was possible to conclude that this was a suicide. Specifically, the sequence of events can be reconstructed as follows: The victim set up the crossbow on two tripods at a suitable height relative to his stature, ensuring stability of the weapon. He then positioned a hollow metal tube in front of the muzzle of the crossbow on an additional tripod, which served as a guide for the trajectory of the bolt to be towards the target. At this point, he removed the safety block from the trigger and positioned himself with his head slightly bent, placing the center of his forehead in front of the hollow metal tube facing the crossbow. By gripping the hooked rod with his right hand the firing mechanism was activated. Upon being struck by the bolt, the victim is likely to have recoiled by a few centimeters and collapsed to the floor against the wall behind him. The metal rod, falling from his hand, is likely to have rolled along the legs of the tripod and came to a stop where it was found.

## Discussion

Over the years, and more generally, in the forensic science community, deaths caused by crossbows have continued to generate intense interest, because of the unique characteristics of the weapon, its historical allure, and the severity of injuries it can inflict. Additionally, crossbows are involved in accidental deaths, homicides, and suicides, and require careful and thorough interpretive evaluation to determine the correct manner of death. From a review of the relevant literature, crossbows have indeed been implicated in some cases of suicide, although self-inflicted injury with this weapon may not be straightforward because of its size and the need to remotely activate the trigger for bolt release. Consequently, in cases where a body is struck by a crossbow bolt, the study of the scene and reconstruction of the crossbow firing mechanism are crucial for correct case assessment. Considering the case under observation, it was deemed appropriate and interesting to conduct a review of the forensic literature on suicides involving crossbows (Table 1). From 1993 to 2023, only 14 relevant reports were published [1, 4, 5, 7–15]. It is noteworthy that all cases involved males ranging in age from 18 to 65 years, with four cases having a documented history of depression (this information was not available for three cases). A particular pattern of target sites emerged, with the head struck in eight cases and the chest in six cases. Thus, a clear preference for highly lethal body regions was observed. Specifically, regarding the head, the main target sites were the temple, eye, mouth, and submental region (this information was not retrievable in two cases), with retained or not retained bolts observed. Regarding the chest, in five cases, the affected region was close to the heart, and only one case involved the right chest. Again, some cases exhibited not retained injuries. Overall, each case involved a single fatal shot, except for one instance in which the individual managed to release two shots successively at the head [7].

**Table 1:** Highlights from the articles concerning crossbow suicide cases during 1993–2023

Year	Ref	Mental illness	Sex	Age	Target	Triggering mechanism	Entry point	Exit point
1994	5		M		Head	information not retrievable	information not retrievable	information not retrievable
	7	no	M	44	Head	Direct	Left eye, palate	retained
1999	8	depression	M	18	Head	Direct	Right temple	Left parietal lobe
		no	M	27	Head	Direct	Below the jaw	retained
2004	9	no	M	38	Head	Direct	Mouth	retained
		no	M	42	Thorax	Direct	Left hemithorax	Back
2009	10	depression	M	49	Thorax	not reported	Left hemithorax	retained
2011	11	not listed	M	58	Thorax	not reported	Left hemithorax	retained
2014	12		M		Head	information not retrievable	information not retrievable	information not retrievable
	13	not listed	not listed	not listed	Head	information not retrievable	Mouth	retained
2017	4	depression	M	40	Head	not reported	Suprahyoid region	Left parietal lobe
2020	14	not listed	M	38	Thorax	information not retrievable	Left hemithorax	not reported
	1	no	M	48	Thorax	not reported	Left hemithorax	Back
2022	15	depression	M	65	Thorax	Indirect with a rod	Right hemithorax	Back

Regarding trigger activation mechanisms, we observed that in almost all cases, the victims pulled the trigger with a finger. However, this type of trigger activation mechanism received relatively little attention in the reports, with vague explanations of the body discovery site and a lack of dynamic reconstruction. At the same time, to determine the nature of the cases, significant importance was given to circumstantial and medical data of the victims, particularly the presence of psychiatric disorders in their medical histories. To the best of our knowledge, there is only one reported case in which crossbow trigger activation occurred indirectly using a modified rod to pull the trigger, resulting in a chest-penetrating injury [15]. This current case differs in that it involved clear premeditation, unlike the other cases in which the deceased individuals personally held the crossbow, aimed at themselves, and directly pulled the trigger with their finger, despite the crossbow being a weapon that is not easily manoeuvred. The current case stands out as a unique finding owing to the distinctive method of carrying out the suicide. It was characterized by meticulous planning to target a specific body location, namely, the center of the forehead. This aspect represents a new finding in the field, as no similar cases involving a crossbow have been recorded. One possible explanation is that the forehead is a small target and it is difficult to hit with a crossbow bolt directly released by the victim. A peculiarity of our case is that the victim used a guiding tube to channel the bolt and ensure that the target was hit. Simultaneously, as the victim was too far from the crossbow trigger to reach it with his hand, a hooked metal rod was used to pull the trigger. Overall, the setup of the equipment and the indirect activation mechanism proved to be extremely effective in carrying out the suicide. This case has also proven to be interesting from a forensic pathological perspective as it provided an opportunity to observe crossbow bolt injuries, which are infrequent. In this case, the short shooting distance resulted in the target being hit at a velocity slightly below the maximum, transferring significant kinetic energy to the body structures, resulting in cranial fracture and extensive brain destruction. The distinctive cranial fracture was noteworthy, as it exhibited notches corresponding to the vanes of the bolthead. These findings can be valuable for forensic pathologists to accurately diagnose similar complex cranial bone injuries, such as when skeletal remains are discovered without the presence of weapons.

Having established that the reported case was based on an indirectly triggered crossbow bolt mechanism for suicidal purposes, we assessed whether there were any reports of similar triggering mechanisms associated with different types of weapons in the forensic field. Overall, suicides planned through indirect activation mechanisms were confirmed to be very rare, particularly those involving sharp force trauma. In this context, the reports were mainly on the use of guillotine-like systems [16–18], activated using ropes. One report describes the use of a speargun triggered by a wooden shaft [19]. More frequently, indirect triggering mechanisms for suicidal purposes have been observed in association with firearm injuries. In this context, they often used homemade weapons triggered by hammer strikes or other types of percussion to launch the projectile [20, 21]. In one case, an artisan-modified trap gun was used, in which a manual and battery-operated illegal ignition device was used to ignite the gunpowder [22]. In another case, a metal tube was used as a cannon for more accurate targeting of the heart, as well as activating the propulsion mechanism [23]. Finally, in one case, a rifle was placed on a wooden support and tied to a nearby tree. Facing the rifle barrel, the victim was positioned, exposing the chest, and the trigger was activated using a lever system pulling a rope [24]. Overall, in these cases, the victim was a middle-aged male, often suffering from a psychiatric disorder, who devised a system aimed at targeting a body site with high lethality [16–24]. Specifically, in cases of sharp-force trauma, the head-neck region was mainly targeted [16–19], in contrast to firearm injuries that involved the head and chest, particularly the heart region [20–24]. Consequently, characteristics very similar to those observed in victims of suicide by crossbow were identified, confirming the requirement for a thorough examination of the body discovery scene to reconstruct event dynamics.

In conclusion, the presented case adds to the limited number of reports in the literature on suicides by crossbow, confirming the typical injury characteristics associated with crossbow bolt wounds and the high potential for harm of this weapon. It also introduces important novelty elements of owing to the uniqueness of the organized system used to precisely target the predetermined location and carry out the suicide. Considering the range of possible fatal suicide scenarios involving a crossbow, it is crucial for forensic pathologists to be prepared to manage these cases in an optimal manner, starting with a careful analysis of the body discovery scene and the triggering mechanism.

## Key points

1. Crossbow injuries are rare, but do occur in forensics.
2. The indirect trigger mechanism of the crossbow bolt is a very rare phenomenon.
3. On-site inspection and reconstruction of events are cornerstones in assessing the manner of death.
4. From 1993 to 2023 there are only 14 reports of suicides with a crossbow in the literature.

## Data Availability

All the data have been reported in the manuscript.

## Code availability

(software application or custom code) Not applicable.
